# Relationship between Serum Cytokeratin-18, Control Attenuation Parameter, NAFLD Fibrosis Score, and Liver Steatosis in Nonalcoholic Fatty Liver Disease

**DOI:** 10.1155/2018/9252536

**Published:** 2018-09-27

**Authors:** Sumitro Kosasih, Wong Zhi Qin, Rafiz Abdul Rani, Nazefah Abd Hamid, Ngiu Chai Soon, Shamsul Azhar Shah, Yazmin Yaakob, Raja Affendi Raja Ali

**Affiliations:** ^1^Gastroenterology and Hepatology Unit, Faculty of Medicine, Universiti Kebangsaan Malaysia, Kuala Lumpur, 56000, Malaysia; ^2^Gastroenterology Unit, Faculty of Medicine, Universiti Teknologi MARA, Shah Alam, Selangor, 40450, Malaysia; ^3^Department of Medical and Health Science, Universiti Sains Islam Malaysia, Nilai, Negeri Sembilan, 71800, Malaysia; ^4^Department of Public Health, Faculty of Medicine, Universiti Kebangsaan Malaysia, Kuala Lumpur, 56000, Malaysia; ^5^Department of Radiology, Faculty of Medicine, Universiti Kebangsaan Malaysia, Kuala Lumpur, 56000, Malaysia

## Abstract

**Backgrounds:**

The aim of this study was to appraise the relationship between serum fragmented cytokeratin-18(CK-18), controlled attenuation parameter (CAP), and liver steatosis assessed by ultrasound (US) in nonalcoholic fatty liver disease (NAFLD) patients.

**Methods:**

Patients who underwent abdominal US were recruited, followed with measurement of CAP using Fibroscan^®^ and serum fragmented CK-18 using enzyme-linked immunosorbent assay. The degree of liver steatosis assessed by US was categorized into mild (S1), moderate (S2), and severe (S3).

**Results:**

A total of 109 patients were included in our study. CAP and fragmented CK-18 level were significantly correlated with liver steatosis grade with r_s_ = 0.56 and 0.68,* p*=0.001, respectively. NAFLD Fibrosis Score was poorly correlated with liver steatosis grade (r_s_=-0.096,* p*=0.318). Using fragmented CK-18 level, area under receiver operating characteristic (AUROC) curves for S≥2 and S≥3 were excellent (0.82 and 0.84, respectively). Using CAP, AUROC curves for detection of S≥2 and S≥3 were good (0.76, 0.77, respectively). We also proposed cut-off value of CAP to detect S≥2 and S≥3 to be 263 and 319db/m, respectively, and fragmented CK-18 level to detect S≥2 and S≥3 (194 and 294 U/L, respectively).

**Conclusions:**

Both the fragmented CK-18 level and the CAP, but not NAFLD Fibrosis Score, were well correlated with hepatic steatosis grade as assessed by US.

## 1. Introduction

Nonalcoholic fatty liver disease (NAFLD) is the liver pandemic in this 21^st^ century, affecting 20-45% population around the world [1–5]. NAFLD has been proven to cause liver fibrosis, liver cirrhosis, and hepatocellular carcinoma [6–9]. Not only does it have an adverse outcome to the liver itself but NAFLD also has been associated with increased rate of metabolic syndrome [10], cardiovascular diseases, and chronic kidney disease [11, 12]. Although NAFLD is usually benign, it may be associated with inflammation and hepatocyte apoptosis resulting in nonalcoholic steatohepatitis (NASH) of 20–30% of subjects. One-fifth of these NASH subjects will progress to develop liver cirrhosis [13].

Liver biopsy is still the gold standard to stage liver fibrosis as it provides a multitude of information on the inflammation activity. However, considering its invasive nature, sampling variability, and cost, other noninvasive modalities of imaging and biomarkers have been developed. The NAFLD fibrosis score (NFS) developed by Angulo et al. utilizes six variables (age, body mass index (BMI), diabetes, aspartatetransaminase (AST), alaninetransaminase (ALT), and albumin) which are commonly available in patient's assessment. It has been shown to reduce the need for biopsy in most NAFLD patients [14]. In NASH, liver cell apoptosis and necroinflammation play a major role. Serum caspase-cleaved fragmented cytokeratin-18(CK-18) reflects the degree of apoptosis and has been shown as an independent predictor in diagnosis of NASH [15–18]. One meta-analysis in 2014 [19] showed area under receiver operating characteristic (AUROC), sensitivity, and specificity of fragmented CK-18 to be 0.84, 0.83, and 0.71, respectively.

For imaging, ultrasound (US) is the first method to be utilized as it is inexpensive, widely available and has a good sensitivity (70-94%) and specificity (70-97%) for liver steatosis [20, 21]. To enhance its sensitivity and specificity, hepatorenal index contrast has been used, resulting in 91% sensitivity and 84% specificity for liver steatosis.

Transient elastography (TE) has been used in several studies to predict steatosis grades in NAFLD patients by using control attenuated parameter (CAP), while stage of fibrosis is measured by liver stiffness measurement (LSM) [22–24]. There are different CAP cut-off values presented by different studies for distinct grades of liver steatosis defined by biopsy (ranging from S0, which indicates no steatosis, to S3, which indicates the highest level of steatosis); for S⩾1(⩾10% of hepatocytes with fat), the CAP cut-off values range from 214 to289dB/m, with a 64%-91% sensitivity range and a 64%-94% specificity range; for S⩾2(⩾33% hepatocytes with fat), the CAP cut-off values range from 255 to 311dB/m, with a 57%-96% sensitivity range and a 62%-94% specificity range; finally, for S3(⩾66% hepatocytes with fat), the CAP cut-off values range from 281 to 310dB/m, with a 64%-100% sensitivity range and a 53%-92% specificity range [23]. A meta-analysis in 2014 [25] showed good pooled sensitivity and specificity for TE in diagnosing fibrosis (F) stage ≥3 (85% sensitivity, 85% specificity) and F4 (92% sensitivity, 92% specificity) and moderate accuracy to predict F≥2 in NAFLD.

The aims of this study are to evaluate the relationship between CAP, LSM, fragmented CK-18, and liver steatosis grade as assessed by US. We also would like to assess the diagnostic performance of CAP and fragmented CK-18 in liver steatosis. Lastly, we aim to compare the level and degree of association of various clinical and laboratory parameters in different liver steatosis grades. This is the first such study in South East Asia.

## 2. Materials and Methods

### 2.1. Patient Characteristics

The study was approved by Universiti Kebangsaan Malaysia (UKM) Ethics Committee and all patients gave written consent prior to participation. We recruited patients, aged more than 18 years old, who underwent ultrasound abdomen between June 2016 and September 2016. Patients with chronic liver disease, pregnancy, malignancy, and excessive alcohol use were excluded.

All recruited patients underwent US abdomen, clinical, laboratory examination, and Fibroscan^®^ assessment.

### 2.2. Clinical Assessment

Comorbid illness (hypertension, diabetes, and dyslipidemia) and alcohol intake, together with anthropometric, laboratory, and past medical history, were obtained from all patients on the same day of ultrasound. Body mass index (BMI) was calculated as body weight in kilograms divided by body height in square meters (kg/m^2^). Waist circumference was measured in a standing position at a level of the umbilicus.

The diagnosis of metabolic syndrome [26–28] was made according to the joint statement of the International Diabetes Federation and World Heart Federation. Excessive alcohol use was defined by an average daily consumption of alcohol of<20g/day for men and <10g/day for women [29].

### 2.3. Ultrasonography

Ultrasound (US) of the abdomen was performed by single experienced consultant radiologist to omit interobserver bias. The degree of liver steatosis on ultrasound was categorized as mild (S1: increased liver echogenicity), moderate (S2: blurring of portal vein branches), or severe (S3: blurring of the diaphragmatic outline) [21, 30–32].

### 2.4. Laboratory Examination

Blood samples were obtained to measure AST, ALT, total cholesterol (TC), triglyceride(TG), high-density lipoprotein cholesterol (HDL-C), low-density lipoprotein cholesterol(LDL-C), fasting blood sugar(FBS), and C-reactive protein(CRP). For measurement of fragmented CK-18, blood as initially processed to plasma and then stored frozen at -80°C. Plasma caspase-3 generated CK-18 fragments were quantitatively measured using the M30 Apoptosense ELISA kit (PEVIVA: Alexis. Grunwald, Germany).

The M30 Apoptosense^®^ ELISA is a solid-phase sandwich enzyme immunoassay. Standards, controls, and samples react with a solid phase capture antibody M5 directed against K18 and the HRP-(horseradish peroxidase) conjugated M30 antibody directed against the K18Asp396 neoepitope. Unbound conjugate is removed by a washing step. TMB Substrate is added. The colour development is stopped and the absorbance is read. The resulting colour is directly proportional to the concentration of the analyte. By plotting a standard curve from known concentrations versus measured absorbance in the microplate reader, the amount of antigen in the sample can be calculated. The concentration of the antigen is expressed as Units per Litre (U/L).

### 2.5. NAFLD Fibrosis Score

This score was calculated based on the study by Angulo et al. [14] with formula of -1.675 + 0.037 xage(years) + 0.094 x BMI(kg/m^2^) +1.13 x diabetes/IFG(yes=1, no=0) + 0.99xAST/ALT ratio – 0.013 x platelet(x 10^9^/L) – 0.66 x albumin(g/dL). Score below -1.455 signified prediction of no advanced fibrosis while score more than 0.676 signified presence of advanced fibrosis, and the score in between is labeled as indeterminate.

### 2.6. Fibroscan

A Fibroscan 502, manufactured by Echosens (Paris, France), was used in the study. We considered results as reliable if interquartile range/median (IQR/M) is less than 30 percent and success rate is over 60 percent. Ten valid fibroscan readings were necessary for an examination to be deemed successful [33]. Controlled attenuation parameter (CAP) was measured to quantify liver steatosis, while the degree of liver fibrosis was displayed as liver stiffness measurement (LSM).

### 2.7. Statistical Analysis

Descriptive statistics were computed for all factors. These were presented in means (M) ± standard error of means (SEM) for normally distributed data, median (Me) with interquartile range(IQR) for nonnormally distributed data, and percentiles and frequencies for categorical factors. Categorical data analysis was performed using Pearson's Chi-Square. Comparison of continuous variables analysis was performed using One-Way ANOVA or Kruskal Wallis where appropriate. The degrees of correlation between parameters and liver steatosis grades were calculated using Spearman's correlation coefficient (r_s_). The predictive value of a variable for detection of liver steatosis was evaluated using AUROC curve analysis. A* p *value of < 0.05 was considered significant. All statistical analyses were performed using SPSS software version 20.0(SPSS Inc. Chicago, IL) for Windows.

## 3. Results

### 3.1. Patients' Characteristic

We recruited 109 patients in this study, with 58 (53.2%) patients were male. Median age of patients in the study was 54, with range from 19 to 78 years of age.

 Several comorbidities were found to be significantly higher in NAFLD patients compared to healthy subjects (Table 1), such as metabolic syndrome (n=56, 82% vs n=12, 29.2%,* p*<0.001), dyslipidemia (n=45, 79% vs n=23, 44.2%, and* p*<0.001), hypertension (n=35, 76% vs n=33, 52.4%, and* p=*0.009), and diabetes (n=40, 78% vs n=28, 48.2%, and* p*=0.001).

### 3.2. Relationship between Liver Biochemistry, CRP, Serum Fragmented CK-18, CAP, LSM and Liver Steatosis Grade

**Figure 1:**
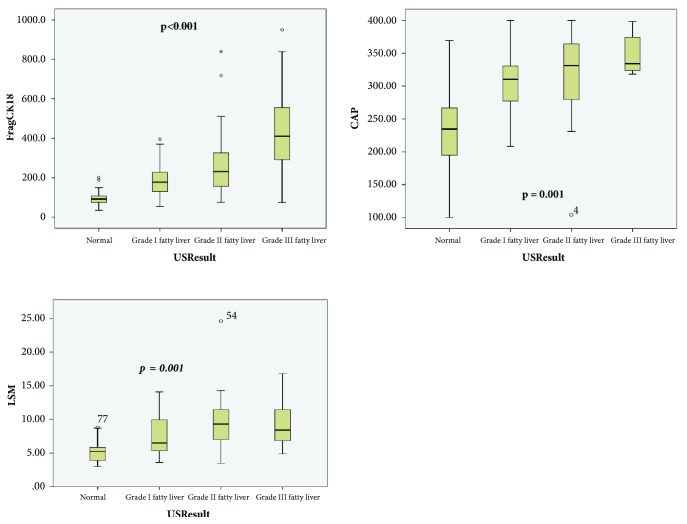
The distribution of CAP, LSM, and CK-18 according to steatosis grade assessed by US. Figure 1 showed significant difference in Fragmented CK-18, LSM, and CAP between nonliver steatosis patients and grades I, II, and III liver steatosis patients.

All liver biochemistry (ALT and AST), as well as apoptotic markers (fragmented CK-18), was significantly higher in NAFLD group compared to healthy subjects (*p*<0.001) (Table 4). Fragmented CK-18 as shown in Figure 1 was significantly different across the healthy subjects, S1, S2, and S3 steatosis at 91(IQR 70-104), 189.5 (IQR 137-227.5), 277 (IQR 189.5-326), and 441(IQR 338-554.5) U/L, respectively.

CAP differed significantly between healthy subjects - 234 (IQR 95-266) dBm and NAFLD group (*p*<0.001) but was not significantly different in between the steatosis grades (S1-310(IQR 277-330), S2 – 331(IQR 279-364), and S3 - 334(IQR 323-374) dB/m).

### 3.3. NAFLD Fibrosis Score and Liver Steatosis

Only two (1.8%) of our patients were categorized as high risk for advanced fibrosis (F3-F4) according to NFS. The remaining were low risk (n=71, 65.1%) and indeterminate (n=36, 33%). We found that NFS was poorly correlated (r_s_=-0.096,* p*=0.318) with liver steatosis grade.

### 3.4. Diagnostic Performance of Fragmented CK-18 and CAP for Assessing Liver Steatosis

**Figure 2:**
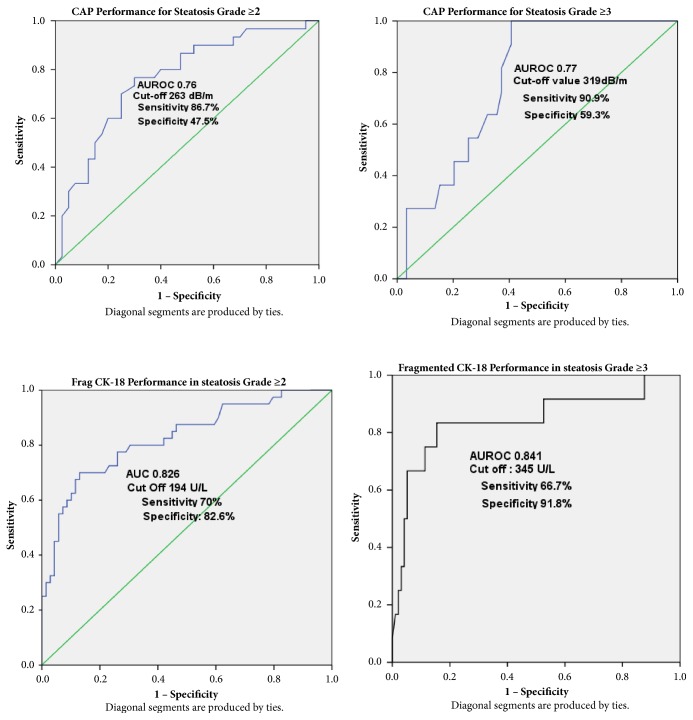
ROC Curve using CAP and Fragmented CK-18, to predict liver steatosis in US. Figure 2 showed CAP value with cutoff value of 263dB/m and 319dB/m had good sensitivity and specificity. FragCK-18 with cut-off value of 194U/L and 345 U/L had a good sensitivity to predict moderate-severe steatosis.

ROC analysis (Figure 2) was performed for all patients (n=109). By using a cut-off value of 194U/L for diagnosis of liver steatosis S≥2, AUROC of fragmented CK-18 was 0.82 [95% Confidence Interval (CI), 0.74-0.91], while sensitivity and specificity were 70% and 82.6%, respectively. By using cut-off value of 294U/L for liver steatosis S≥3, AUROC was 0.84 (95%CI, 0.69-0.99), while sensitivity and specificity were 75%, and 87.6%, respectively.

CAP has demonstrated satisfactory diagnostic performance in detecting liver steatosis. AUROC for liver steatosisS≥2 by using cut-off value of 263dB/m was 0.76(95%CI, 0.65-0.88), with sensitivity of 86.7% and specificity of 47.5%, while AUROC for liver steatosis S≥3 (with a cut-off value of 319 dB/m) was 0.77(95%CI, 0.65-0.88), with sensitivity of 90.9% and specificity of 59.3%.

### 3.5. Factors Associated with CAP

Using univariate linear regression analysis, BMI (*β*=5.5,* p*=0.001), FBS (*β*=13.07,* p*=0.02), HDL-C (*β*=-76.35,* p*=0.004), waist circumference (*β*=5.8,* p*=0.001), fragmented CK-18 (*β*=0.17,* p*=0.001), LSM(*β*=10.1,* p*=0.001), and steatosis grade (*β*=36.2,*p*=0.001) were associated with CAP in all our patients. Among all these factors, only LSM, TG, and steatosis grade were shown to be independent factors related to CAP in multivariate linear regression analysis (Table 5). With every increment of LSM of 1kPa and 1 mmol/L of TG, CAP score would increase by 7.9dB/m and 21.2dB/m, respectively, while CAP scores would increase by 31.9dB/m with every increment in liver steatosis grade.

## 4. Discussion

Liver biopsy is considered the gold standard for assessing the degree of hepatic steatosis and fibrosis; however, biopsy is rarely done due to its risk and limitation. Liver biopsy has several limitations such as small area of examination (only representing 1/50000 of whole liver), sampling variabilities and error, inter- and intraobserver variability [34–36]. Therefore, steatosis and fibrosis are now being more commonly assessed by using noninvasive modalities like imaging and biomarkers. The short examination time and noninvasiveness make abdominal ultrasonography the best initial screening method for NAFLD. CAP and LSM in fibroscan is a recent, novel way to diagnose NAFLD as well as quantifying hepatic steatosis and fibrosis accurately and in a convenient manner. Fragmented CK-18 is a biomarker that currently under investigation to diagnose NASH and assess the degree of fibrosis [15].

In our study, we found that patient with hypertension, dyslipidemia, diabetes, and metabolic syndrome had higher proportion of suffering from NAFLD, regardless of age and sex. We also found that higher BMI, waist circumference (Table 2), and liver biochemistry (ALT, AST≥35U/L) (Table 3) are associated with increased severity of liver steatosis, as assessed by US. These findings are consistent with previous studies [37, 38]. Chia et al. showed that there was a significant difference in ALT and AST values between mild and significant fatty liver, although they used broader definition of fatty liver population, which included non-NAFLD patients as well.

In a study by Angulo et al. [14], NFS was shown to have a high positive predictive value (90%) of diagnosing advanced fibrosis. However, in our study, we concluded that ultrasonography is not a good tool to differentiate degree of fibrosis, since many of our patients were categorized into low and indeterminate groups, although US showed steatosis grade≥2. This is consistent with previous studies that suggested it is difficult to differentiate steatosis and fibrosis, as they may have the same echographic appearance in hepatic US [32].

In our study, fragmented CK-18 level showed a good correlation with steatosis grade as assessed by US with r_s_ of 0.68. One study by Tsutsui [16] showed correlation of fragmented CK-18 with steatosis assessed by histology activity is 0.482. This discrepancy of result could be explained by possible smaller area of steatosis assessed by liver biopsy. Fragmented CK-18 although was known as a necroinflammatory and apoptotic marker, in our study, we believed that CK-18 has a good predictive value of detecting liver steatosis as assessed by US, especially in liver steatosis grade≥2 and ≥3, with AUROC 0.82, 0.84, respectively.

We had demonstrated a moderate correlation between CAP, LSM, and the degree of liver steatosis (r_s_=0.56, r_s_=0.49, respectively). Our CAP correlation coefficient (r_s_=0.56) was a little bit lower than that of previous study by Carvalhana (r_s_ = 0.73,* p*<0.001) [31] but comparable to that of the recent study by Ahn JM et al. (r_s_ =0.58,* p*<0.001) [39, 40]. This discrepancy of results may be explained by differences in both studies populations, by Carvalhana et al. and Ahn JM et al., in which they included chronic viral hepatitis and alcoholic liver disease as well as using the different classification criteria of steatosis grades on US examination.

The predictive value of CAP to detect liver steatosis was compared with previous studies conducted. We demonstrated that optimal cut-off values for detecting S≥2 and S≥3 steatosis by using Youden index were 263 dB/m and 319dB/m, respectively, and these were comparable with previous studies done in Canada and France [40, 41], with cut-off value of 250db/m and 317db/m for S≥2 and S≥3 reported, respectively. However, our cut-off values differed from those in other studies [42–44]. These differences might be explained by different study populations as some studies include not only NAFLD but also chronic viral hepatitis patients.

In our study, predictive value of fragmented CK-18 to detect liver steatosis grade as assessed by US was comparable to studies conducted previously. However, majority of the studies conducted were to detect steatohepatitis (NASH) rather than simple NAFLD on liver biopsy [18, 45, 46].

Our study also showed that liver steatosis grade detected by ultrasound, LSM, and TG was independently associated with CAP. However, study by Ahn et al. showed that only US liver steatosis grade independently affected the CAP score [39]. This could be explained by their different study population which included alcoholic liver disease patients. None of the necroinflammatory markers such as ALT, AST, CRP, and fragmented CK-18 were independently associated with CAP. All these findings were quite consistent with other previous studies. [39, 41].

The strength of this study was its ability to show the relationship of CAP (a convenient but uncommonly used tool), with US (the most commonly available tool) for assessing NAFLD patients. In addition, we compared this relationship with fragmented CK-18, a new biomarker, in the NAFLD patients.

However, our study had several limitations. First, steatosis grade assessed by US has a limitation due to its subjective interpretation. Secondly, single operator US may cause a bias in the result. Thirdly, we did not have a comparison against liver biopsy, regarded as gold standard as a reference for our US findings.

## 5. Conclusion

In conclusion, our study showed fragmented CK-18 and CAP were relatively well correlated with steatosis grade as assessed by US. NAFLD fibrosis score, however, did not show any correlation with US. We proposed the cut-off values for fragmented CK-18 and CAP in moderate and severe liver steatosis; fragmented CK-18 for S≥2 and S≥3 were 194U/L and 345U/L, respectively; CAP for S≥2 and S≥3 were 263dB/m and 319dB/m, rspectively. However, larger scale studies are needed to confirm the optimal cut-off values.

## Figures and Tables

**Table 1:** Patients' characteristics and comorbid conditions. NAFLD: nonalcoholic fatty liver disease; BMI: Body Mass Index; SEM: Standard Error of Means.

	Non-hepatic steatosis n=41	NAFLD n = 68	*p* value
Gender	21:20	37:31	
(Male: Female)			
Age, years			
Mean ±SEM	50.4±2.3	53.1±1.5	0.231
BMI, kg/m^2^			
Mean ± SEM	24.6±0.89	29.05±0.60	0.000
Diabetes	11 (26.8%)	40 (78.4%)	0.001
Hypertension	11 (26.8%)	35 (76%)	0.009
Dyslipidemia	12 (29.2%)	45 (78.9%)	<0.001
Metabolic Syndrome	12 (29.2%)	56 (82.4%)	<0.001

**Table 2:** Relationship between anthropometric data with liver steatosis as assessed by US. BMI: Body Mass Index; M: means; SEM: standard error or means.r_s_: Spearman coefficient correlation; *∗*: significant.

	Non Liver steatosis (M±SEM)	LiverSteatosis Grade			*p* value	r_s_
		S1 (M±SEM)	S2 (M±SEM)	S3 (M±SEM)		
BMI, kg/m^2^	24.65±0.88	27.73±0.99	29.96±0.90	29.99±1.02	<0.001	0.46*∗*
Waist Circumference, inch	33.40±0.78	35.42±0.98	37.86±0.86	37.95±0.72	0.001	0.40*∗*

**Table 3:** Relationship between laboratory data and liver steatosis grade as assessed by US. HDL: high-density lipoprotein; FBS: fasting blood sugar; M: mean; SEM: standard error of means; r_s_, Spearman coefficient correlation; *∗*: significant.

	Non hepatic-steatosis (M±SEM)	Hepatic Steatosis Grade			*p* value	r_s_
		S1 (M±SEM)	S2 (M±SEM)	S3 (M±SEM)		
HDL, mmol/L	1.29±0.05	1.31±0.07	1.16±0.04	1.13±0.06	0.181	-0.14
Triglyceride mmol/L	1.50±0.13	1.50±0.12	1.53±0.11	1.83±0.19	0.574	0.15
Total Cholestrol, mmol/L	4.88±0.16	4.95±0.23	4.78±0.21	5.01±0.30	0.920	-0.05
FBS, mmol/L	6.52±1.01	8.15±1.74	6.86±0.43	6.49±0.44	0.738	0.35
Albumin, gr/dL	40.68±0.58	40.8±0.45	41.1±0.49	40.3±0.82	0.875	0.01
Platelet, x10^9^/L	251.4±10.46	245.8±11.8	272.9±8.92	259.4±10.82	0.325	0.11

**Table 4:** Relationship between inflammatory markers, CK-18, fibroscan findings, and liver steatosis grade as assessed by US. Me, Median; r_s_: Spearmancoefficient correlation; *∗*: significant. ALT: alanine transaminase; AST: aspartate transaminase; CRP: C-reactive protein; CK-18: cytokeratin-18; CAP: controlled attenuated parameter; LSM: liver stiffness measurement.

	Non hepatic-steatosis Me(IQR)	Hepatic Steatosis Grade			*p* value	r_s_
		S1 Me(IQR)	S2 Me(IQR)	S3 Me(IQR)		
ALT, U/L	18.5(14-27.5)	31(21.75-65)	56(31-76)	68(31-112)	<0.001	0.54*∗*
AST, U/L	20.5(17-24.5)	22.5(21-34.5)	28(24-47.5)	45(34.5-48.5)	<0.001	0.53*∗*
CRP, mg/dL	0.08(0.05-0.2)	0.23(0.06-0.45)	0.27(0.09-0.73)	0.33(0.08-0.43)	0.462	0.25
CK-18, U/L	91(70-104)	189.5(137-227.5)	277(189.5-326)	441(338-554.5)	<0.001	0.68*∗*
CAP, dB/m	234(95-266)	310(277-330)	331(279-364)	334(323-374)	<0.001	0.56*∗*
LSM, kPa	5.3(3.9-5.9)	6.5(5.4-9.9)	9.3(7-11.5)	8.4(6.9-11.5)	0.001	0.49*∗*

**Table 5:** Factors Influencing CAP (controlled attenuation parameter) in fibroscan. Using Univariate and Multivariate Linear Regression Analysis. Β: beta coefficient. Uni: univariate regression analysis; Multi: multivariate regression analysis. BMI: Body Mass Index; FBS: fasting blood sugar; HDL: high-density lipoprotein; TG: triglyceride; TC: total cholesterol; ALT: alanine transaminase; AST: aspartate transaminase; CRP: C-reactive protein; CK18: cytokeratin-18; LSM: liver stiffness measurement; US: ultrasonography; *∗*: significant.

Variable	Uni		Multi	
	***β***	*p* value	***β***	*p* value
Age	0.42	0.53	-.505	0.442
BMI	5.5	0.001	-0.811	0.834
FBS	13.07	0.02	7.54	0.064
HDL	-76.35	0.004	-26.3	0.337
TG	16.6	0.112	21.2	0.04*∗*
TC	-7.3	0.339	-9.4	0.215
WaistCircum	5.8	0.001	1.9	0.612
ALT	0.47	0.07	-0.17	0.588
AST	0.92	0.04	-0.69	0.362
Albumin	-0.69	0.81	-0.06	0.98
CRP	24.9	0.228	-13.4	0.533
Platelet	0.09	0.58	-0.044	0.791
CK18	0.172	0.001	-0.052	0.394
LSM	10.1	0.001	7.9	0.002*∗*
USResult	36.2	0.001	31.9	0.003*∗*

## Data Availability

The data used to support the findings of this study are available from the corresponding author upon request.

## Appendix

See Tables 1–5 and Figures 1 and 2.
